# Evidence from routine clinical practice: EMPRISE provides a new perspective on CVOTs

**DOI:** 10.1186/s12933-019-0920-3

**Published:** 2019-08-31

**Authors:** Guntram Schernthaner, Avraham Karasik, Agnė Abraitienė, Alexander S. Ametov, Zsolt Gaàl, Janusz Gumprecht, Andrej Janež, Susanne Kaser, Katarina Lalić, Boris N. Mankovsky, Evgeny Moshkovich, Marju Past, Martin Prázný, Gabriela Radulian, Lea Smirčić Duvnjak, Ivan Tkáč, Kārlis Trušinskis

**Affiliations:** 1 Medical University of Vienna, Vienna, Austria; 2 Sheba Medical Center and Tel Aviv University, Tel Aviv, Israel; 3 Clinic of Internal Diseases, Family Medicine and Oncology, Institute of Medicine, Faculty of Medicine, Vilnius University Hospital Santaros Klinikos, Vilnius University, Vilnius, Lithuania; 4 Russian Medical Academy for Continuous Professional Education, Ministry of Education of the Russian Federation, Moscow, Russia; 5 Department of Medicine, András Jósa Teaching Hospital, Nyíregyháza, Hungary; 6 Medical University of Silesia, Katowice, Poland; 7 Department of Endocrinology, Diabetes and Metabolic Diseases, University Medical Centre, Ljubljana, Slovenia; 8 Department of Internal Medicine I and CD Laboratory for Metabolic Crosstalk, Medical University of Innsbruck, Innsbruck, Tirol Austria; 9 Clinic for Endocrinology, Diabetes and Metabolic Diseases, Clinical Centre of Serbia, Faculty of Medicine, University of Belgrade, Belgrade, Serbia; 10 National Medical Academy for Postgraduate Education, Kiev, Ukraine; 11 Diabetes and Endocrinology Clinic, Clalit Medical Services, Ramat Gan, Israel; 12 Estonian Diabetes Center, Tallinn, Estonia; 13 3rd Department of Internal Medicine, 1st Faculty of Medicine, Charles University in Prague, Prague, Czech Republic; 14 Carol Davila University of Medicine and Pharmacy, Bucharest, Romania; 15 School of Medicine, University of Zagreb, Vuk Vrhovac University Clinic-UH Merkur, Zagreb, Croatia; 16 Department of Internal Medicine 4, Faculty of Medicine, Safarik University in Košice, Košice, Slovakia; 17 Latvian Center of Cardiology, Stradiņš Clinical University Hospital, Rīga Stradiņš University, Riga, Latvia

**Keywords:** Type 2 diabetes, CVOTs, Real-world evidence, EMPRISE, EMPA-REG OUTCOME, Heart failure

## Abstract

EMPA-REG OUTCOME is recognised by international guidelines as a landmark study that showed a significant cardioprotective benefit with empagliflozin in patients with type 2 diabetes (T2D) and cardiovascular disease. To assess the impact of empagliflozin in routine clinical practice, the ongoing EMPRISE study is collecting real-world evidence to compare effectiveness, safety and health economic outcomes between empagliflozin and DPP-4 inhibitors. A planned interim analysis of EMPRISE was recently published, confirming a substantial reduction in hospitalisation for heart failure with empagliflozin across a diverse patient population. In this commentary article, we discuss the new data in the context of current evidence and clinical guidelines, as clinicians experienced in managing cardiovascular risk in patients with T2D. We also look forward to what future insights EMPRISE may offer, as evidence is accumulated over the next years to complement the important findings of EMPA-REG OUTCOME.

## Introduction

Cardiovascular (CV) outcomes are now recognised by international guidelines as an important consideration in treatment choice for patients with type 2 diabetes (T2D) and CV disease (CVD) [1–6]. This is an exciting development, which follows the discovery from CV outcomes trials (CVOTs) that some antidiabetic agents have a cardioprotective effect in at-risk patients [7–10].

The first CVOT to show cardioprotection was EMPA-REG OUTCOME, in which empagliflozin rapidly reduced the risk of hospitalisation for heart failure (HHF) and CV death compared with placebo, independently of glycaemic control [11]. Despite the clinical importance of this finding, the underlying mechanism (or mechanisms) remains a matter of speculation and debate, with roles postulated for processes ranging from inflammation, oxidative stress and ionic exchange in the myocardium [12] to blood viscosity and wall shear stress in the carotid arteries [13].

CVOTs have now been completed for multiple agents in the SGLT2 inhibitor, GLP-1 receptor agonist and DPP-4 inhibitor classes; among these, cardioprotective effects have been reported for all SGLT2 inhibitors investigated (although not consistently across different outcomes) and, in addition, for some GLP-1 receptor agonists [14]. Such paradigm-shifting data can pose a challenge for clinicians, who must integrate learnings from a proliferating number of clinical studies into routine clinical practice, where patients and conditions are typically more diverse than the tightly controlled cohorts seen in randomised controlled trials (RCTs) such as CVOTs. Efforts are now underway to collect real-world evidence (RWE) that may help to bridge this gap, providing insights into how beneficial CV effects seen in CVOTs are reflected in real-world populations and everyday clinical decision-making scenarios [15].

EMPRISE is an ongoing RWE study of data collected from US healthcare databases, comparing outcomes in patients newly initiated with empagliflozin vs DPP-4 inhibitors [16]. The study will complement the findings of EMPA-REG OUTCOME with routine clinical practice data that encompasses a more diverse patient population, including a broader spectrum of CV risk, and an active comparator that prescribers currently use in a similar position to empagliflozin in the treatment pathway [16]. Over 5 years, EMPRISE will enrol approximately 200,000 patients, and generate insights on a wide range of effectiveness, safety and health economic outcomes [16].

Recently, a first interim analysis of EMPRISE was published, covering HHF outcomes among ~ 35,000 patients in the time period from August 2014 through September 2016 (~ 33,000 in the primary analysis, which looked only at a single DPP-4 inhibitor, sitagliptin) [16]. Despite a short mean follow-up time of 5.3 months in the early results to emerge from EMPRISE, the data are very encouraging in confirming a HHF benefit in patients receiving empagliflozin [16].

We recently convened as a group of experts from Central and Eastern Europe to discuss the newly described EMPRISE study, together with the results of its first interim analysis, from the perspective of our own knowledge and clinical experience on managing CV risk in patients with T2D. In this Commentary article, we summarise our discussions, considering the role for RWE in supporting CVOTs in clinical decision making, and placing the findings from the first interim EMPRISE analysis into the context of EMPA-REG OUTCOME, other SGLT2 inhibitor CVOTs and international guidelines. We believe that these insights will be useful for clinicians from our region and beyond who wish to assess the evidence for optimising treatment for their patients in routine clinical practice.

## RWE as a complement to CVOTs

**Fig. 1:**
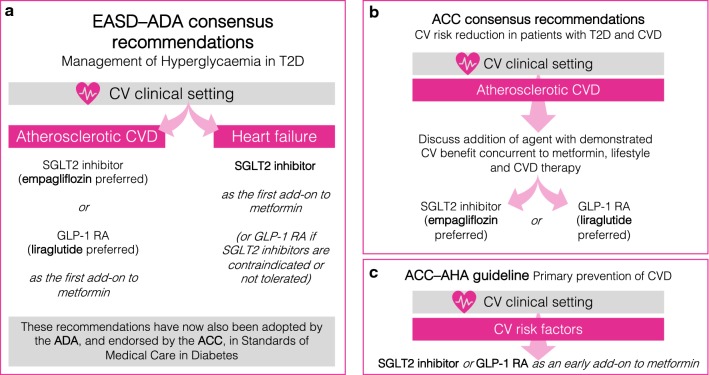
SGLT2 inhibitors—what do guidelines say? **a** The EASD and the ADA jointly published a position statement on the management of hyperglycaemia in patients with T2D that were updated to reflect evidence from CVOTs. The revised treatment pathway, which recommends an early consideration of CV risk, is also now incorporated into the ADA’s Standards of Medical Care in Diabetes, which is for the first time endorsed by the ACC. Within a CV setting, the guidelines distinguish between atherosclerotic CVD, where empagliflozin or liraglutide are preferred as 2nd line to metformin, and HF, where any SGLT2 inhibitor is preferred in this position. **b** The ACC has recently published its own consensus pathway for CV risk reduction in patients with T2D and CVD, advising that agents with proven CV benefit are considered concurrently to metformin, with a preference for empagliflozin or liraglutide. **c** A separate guideline developed by the ACC jointly with the AHA addresses the primary prevention of CVD. In patients with T2D and CV risk factors, an SGLT2 inhibitor or GLP-1 RA is recommended as an early add-on to metformin. The guideline highlights evidence from CVOTs suggesting primary prevention of HF with SGLT2 inhibitors. *ACC* American College of Cardiology, *ADA* American Diabetes Association, *AHA* American Heart Association, *CV* cardiovascular, *CVD* CV disease, *CVOT* CV outcomes trial, *EASD* European Association for the Study of Diabetes, *GLP-1 RA* glucagon-like peptide-1 receptor agonist, *HF* heart failure, *SGLT2* sodium–glucose transporter 2, *T2D* type 2 diabetes

SGLT2 inhibitor CVOTs such as EMPA-REG OUTCOME have yielded impressive results, with HHF reductions across the class and CV death outcomes with empagliflozin carrying sufficient weight to influence major international diabetology and cardiology guidelines [1–6]. New recommendations guided by CVOTs include an early consideration of CV risk and preference for an agent with proven CV benefit as a first-add on to metformin in an atherosclerotic CVD setting (preferring empagliflozin or liraglutide) [1–3, 6] or an SGLT2 inhibitor as a first add-on to metformin in a HF setting [1–3, 5] (Fig. 1). However, numerous gaps in our knowledge of CV outcomes in T2D remain, and we believe that RWE studies can have a role here, by complementing CVOTs with supporting evidence where data generation in a RCT would not be feasible. It is welcome that several completed and ongoing studies are now providing such opportunities to complement CVOTs with RWE on SGLT2 inhibitors [17].

### EMPRISE as a complementary study to EMPA-REG OUTCOME

EMPRISE is a large-scale RWE study specifically undertaken to assess whether the CV effectiveness of empagliflozin observed in EMPA-REG OUTCOME [7] can be confirmed in routine clinical practice [16]. The study uses propensity score (PS) matching to compare patients newly initiated on empagliflozin with those newly initiated on a DPP-4 inhibitor, which echoes a treatment choice often faced in the management of T2D [16].

Data collection is currently ongoing from 3 large US databases (from two commercial insurers and Medicare fee-for-service), with a target cohort size of ~ 200,000 patients over 5 years by study completion [16]. Each database has a different strength: MarketScan provides the largest number of patients; Optum is the most enriched for records with laboratory data; and Medicare mainly represents elderly patients, who have low commercial insurance coverage [16, 18]. PS matching ensures that outcomes are captured between comparable patients, with each individual in the empagliflozin arm matched with a counterpart in the comparator arm using more than 140 covariates [16] (Table 1).

**Table 1:** Steps to minimise confounding in the EMPRISE study design

***Minimising confounding*** Key aspects of the EMPRISE study design	
*PS matching*	Patients are 1:1 matched with a “nearest neighbour” based on 140 predefined baseline characteristics (“covariates”) Covariates include key factors relating to disease severity (such as # antidiabetic medications), comorbidities (such as CVD history) and many other clinical and demographic characteristics
*Appropriate comparator choice*	The most commonly prescribed DPP-4 inhibitor is the chosen active comparator to empagliflozin, owing to the similar position of DPP-4 inhibitors to SGLT2 inhibitors in the treatment pathway Using a comparator with a similar position is designed to maximise the similarity of disease severity between cohorts
*No overlap between comparators*	Patients are excluded if they had received any SGLT2 inhibitor or DPP-4 inhibitor in the year preceding cohort entry, and follow-up is terminated if a patient switches to the comparator Minimises the potential for immortal time bias
*Sequential enrolment*	PS matching is performed independently for each enrolment Ensures that study arms are balanced not just across the full cohort, but also for temporally matched populations
*“As-treated” approach*	Follow-up captures only outcomes occurring during treatment exposure + 30 days Minimises bias from confounding events not related to treatment
***Assessing balance between cohorts*** Data used to independently confirm robustness of PS matching approach	
*Baseline laboratory scores*	A range of laboratory scores at baseline are available for a subset of the population, including Hb1Ac, cholesterol and creatinine levels These scores are not used for PS matching, and so can provide an independent indication of equivalence between study arms
**Sensitivity analyses** In each case, the conclusions regarding HHF benefit with empagliflozin were unchanged	
*High-dimensional PS matching*	PS matching with 100 additional covariates
*Alternative comparator*	The sitagliptin cohort is replaced with a cohort composed of patients receiving any DPP-4 inhibitor
*Subgroup analyses*	Subgroup analyses include: With/without CVD at baseline With/without HF at baseline Gender Empagliflozin dose
*Alternative HHF definition*	Broadening the definition of HHF from hospitalisation with HF in the primary discharge position to hospitalisation with HF in any discharge position
*Control outcome*	An outcome with an expected null finding (flu vaccination)

The EMPRISE study design used several approaches to minimise confounding [16], although undetected bias from residual confounding cannot be excluded. *CVD* cardiovascular disease, *DPP-4* dipeptidyl peptidase-4, *HF* heart failure, *HHF* hospitalisation for HF, *PS* propensity score, *SGLT2* sodium–glucose transporter 2

We see several benefits to EMPRISE as an opportunity to generate evidence that is beyond the scope of CVOTs: outcomes in a more diverse patient population (both with and without clinical evidence of CVD); a comparator that is more relevant than placebo to clinical practice (DPP-4 inhibitors, in keeping with a treatment choice we commonly face in our clinical practice); health economic outcomes; and a larger cohort for the study of safety outcomes [16].

We recognise that extensive efforts have been made in the study design to avoid bias and minimise confounding (Table 1), although it must be emphasised that residual confounding cannot be excluded, as treatment choices are open label and non-randomised [16]. One possible source of bias that has been debated as a factor in previous RWE studies with SGLT2 inhibitors is the potential phenomenon of immortal time bias [19–21], which may occur when a different positioning in the treatment pathway is not accounted for in the study design. However, EMPRISE convincingly addresses these concerns in three ways. First, all patients who had previously taken either class of agent (SGLT2 inhibitor or DPP-4 inhibitor) were excluded [16]. Second, an active comparator was chosen (sitagliptin) that is similarly positioned to empagliflozin in the treatment pathway [16]. Third, PS matching included relevant variables to control for immortal time bias, such as the number of previous antidiabetic medicines and comorbidity score [16].

## The EMPRISE study—what have we learned so far?

The planned interim analysis of EMPRISE that was recently published covers data on HHF events with empagliflozin vs sitagliptin from August 2014 through September 2016, with a mean of 5.3 months follow-up [16]. The number of patients included in the analysis, after PS matching, was 16,443 for each treatment arm [16].

### A balanced study population with a broad spectrum of CV risk

An assessment of standard deviation showed that baseline characteristics between study arms were well balanced [16], including various CV risk factors (Table 2). The number of previous antidiabetic medicines was equivalent between arms before, as well as after, PS-score matching, showing that both agents were typically used third line [16].

**Table 2:** Key baseline characteristics in the 8/2014–9/2016 EMPRISE cohort

	Before PS matching			After PS matching	
	Sitagliptin (N = 201,839)	Empagliflozin (N = 18,880)		Sitagliptin (N = 16,443)	Empagliflozin (N = 16,443)
Diabetes medication			⟶ PS matching		
# antidiabetic drugs (mean)	2.2	2.3		2.2	2.2
Treatment naïve (%)	13%	7%		8%	8%
CV risk factors					
Any CVD (%)	37%	24%		25%	25%
CAD (%)	26%	18%		18%	18%
Stroke (%)	10%	5%		6%	6%
PAD (%)	10%	5%		5%	5%
HF (%)	11%	5%		5%	5%
Lab results (not used for PS matching)					
HbA1c (mean)	8.3	8.5		8.6	8.5

Baseline characteristics confirmed the success of creating balanced study arms in the first interim EMPRISE analysis [16]. Cohorts had equivalent scores for a wide range of factors, including CV risk factors; shown here are scores for some key characteristics of interest. Treatment history was included in the PS score to ensure that treatment position was considered during matching. However, the similar treatment histories and HbA1c scores even prior to PS matching confirm that the active comparator was appropriately chosen as in an equivalent position in the treatment pathway to empagliflozin. *CAD* coronary artery disease, *CV* cardiovascular, *CVD* CV disease, *HF* heart failure, *PAD* peripheral artery disease, *PS* propensity score

Laboratory results were available for only ~ 20% of patients [16], which we see as a limitation. However, key baseline characteristics in this subset of patients were well matched between study arms, including HbA1c, creatinine, cholesterol, LDL and HDL [16], even though these laboratory results were not used for PS matching. Therefore, the covariates used in PS matching seem to have ensured well-balanced metabolic profiles across the full cohort, as expected from a previous study using the same PS-matching methodology [22]. The Hb1Ac balance between cohorts was further confirmed by the similar levels seen between study arms even prior to PS matching [16].

The spectrum of CV risk was substantially broader than in CVOTs such as EMPA-REG OUTCOME, with 75% of patients with no reported history of CVD at baseline [7, 16] (Table 2; in EMPA-REG OUTCOME, all patients had CVD at baseline, as stipulated in inclusion criteria [7]). Owing to PS matching, baseline rates remained equivalent between study arms even when looking at individual components of CV risk, with coronary artery disease (CAD) the most prevalent (18%). Baseline prevalence of heart failure was 5%, approximately half the value reported in EMPA-REG OUTCOME, although the identification of heart failure may have been inconsistent between studies [7, 16].

### Results consistent with EMPA-REG OUTCOME—but in a broader patient cohort

**Fig. 2:**
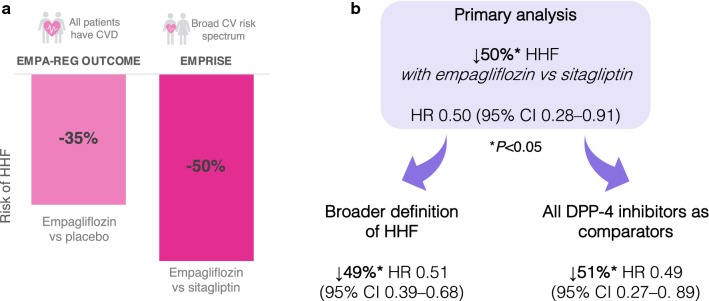
HHF events in EMPRISE and EMPA-REG OUTCOME. **a** The relative risk reduction of HHF in the first interim analysis of EMPRISE was consistent with that seen in EMPA-REG OUTCOME, confirming the robustness of empagliflozin’s HHF benefit in routine clinical practice, in a population with a broader CV risk profile, and against a DPP-4 inhibitor as an active comparator. **b** The first interim analysis of EMPRISE had a primary analysis of hospitalisation events with discharge diagnosis of HF in the primary position compared between empagliflozin and sitagliptin. However, secondary analyses showed that the HHF benefit with empagliflozin was robust even when using a broader definition of HHF (discharge diagnosis of HF in any position) or broadening the active comparator to include all DPP-4 inhibitors, not just sitagliptin. *CV* cardiovascular, *DPP-4* dipeptidyl peptidase-4, *HF* heart failure, *HHF* hospitalisation for HF

A comparison of HHF event rates between PS-matched treatment arms showed a 50% reduction in relative risk with empagliflozin vs sitagliptin (HR 0.50; 95% CI 0.28–0.91) (Fig. 2a) [16]. We expected to see a reduction given the events reported for empagliflozin vs placebo in EMPA-REG OUTCOME (HR 0.65; 95% CI 0.50–0.85), but it was reassuring to see how consistent this effect was in a real-world setting and with a more diverse patient population (Fig. 2a) [7, 16].

We were also encouraged to see such a robust result in a first interim analysis. Although already sufficiently powered for studying HHF events, the number of patients (32,886) is far smaller than the anticipated 200,000 by study completion [16]. Therefore, future analyses will provide even more evidence on HHF outcomes, and also be powered to shed light on rarer outcomes. The rapid emergence of an effect on HHF with empagliflozin, after a mean follow-up time of 5.3 months, is also consistent with EMPA-REG OUTCOME, where CV benefits vs placebo, including reduction in HHF, were apparent early in the study [11].

EMPRISE has provided an important opportunity to observe CV outcomes with empagliflozin in patients without a history of CVD at baseline, who were not included in EMPA-REG OUTCOME [16]. As a first interim analysis, the study is not yet powered for detailed subgroup analyses, but current results do strongly point to a HHF benefit with empagliflozin that is consistent between patients with and without CVD at baseline [16]. We must therefore consider that the clinical and cost implications of reducing HHF with empagliflozin may extend beyond the patient profile of EMPA-REG OUTCOME to also include those without clinical evidence of CVD.

### How robust is the HHF benefit with empagliflozin?

To ensure that the study design has not biased RWE findings, it is important to ensure that conclusions are robust to alternative parameter selections and inclusion criteria. Additional analyses of the interim EMPRISE data showed that including all DPP-4 inhibitor agents in the comparator arm or using a broader definition for HHF did not have a meaningful effect on outcomes [16] (Fig. 2b). These additional analyses provide reassurance that the conclusion of HHF benefit with empagliflozin vs DPP-4 inhibitors is robust.

## EMPRISE in context—where does RWE fit in the bigger CVOT picture?

The key CV benefits shown in EMPA-REG OUTCOME were reduced risks of CV death and HHF [7]. More broadly, SGLT2 inhibitor CVOTs have consistently shown a reduced risk of HHF vs placebo, whereas a reduction in CV death has thus far been unique to empagliflozin [7–9]. Consequently, important questions remain about class effect with SGLT2 inhibitors that RWE may help to address.

### Heart failure

EMPRISE adds to accumulating evidence from SGLT2 inhibitor CVOTs that HF benefits include primary as well as secondary prevention [7–9]. These findings are also consistent with the recent CREDENCE trial of canagliflozin in patients with T2D and chronic kidney disease (CKD) [23], and earlier RWE studies that compared SGLT2 inhibitors as a class with all other glucose-lowering drugs [24–26].

The effect size observed across all studies is consistently impressive, typically 30–40% in the RCTs [7–9, 23] and 40–50% in the RWE studies [16, 24, 25]. Furthermore, these studies collectively show that HHF is reduced in a broad spectrum of patients with T2D, consistently including those without a prior history of HF. Therefore, there is considerable and wide-ranging evidence for substantial primary and secondary prevention of HHF. As such, new American Heart Association (AHA)–American College of Cardiology (ACC) guidelines on the primary prevention of CVD recommend SGLT2 inhibitors as an early add-on to metformin in patients with T2D and CV risk factors (Fig. 1c), citing HHF reductions reported in CVOTs even for primary prevention populations [5].

HHF events in EMPA-REG OUTCOME occurred in a less diverse population than is seen for real-world HHF events [27]. EMPRISE is now providing insights into HHF outcomes in a broader population with T2D, while numerous ongoing RCTs will assess SGLT2 inhibitors specifically in a HF setting [28], including in patients without T2D, and we look forward to understanding more about the impact of these agents on HF once data become available.

### CV death

While HHF benefit seems to be a consistent observation with SGLT2 inhibitors in patients with T2D, empagliflozin remains the only SGLT2 inhibitor proven to reduce CV death (38% reduction in EMPA-REG OUTCOME [7]). Although canagliflozin did show a trend towards a reduction in both the CANVAS Program and CREDENCE, this did not meet significance [8, 23], while there was no apparent effect with dapagliflozin in DECLARE-TIMI 58 [9]. Empagliflozin is also the only agent in the class proven to reduce death by any cause, with a 32% reduction in EMPA-REG OUTCOME, but this is unsurprising given that the CV death component was the main driver of this benefit [7].

We do not yet know whether the inconsistent CV death outcomes between agents is due to intrinsic differences in treatment effects or differences in patient populations and study designs, such as the CV risk profile at baseline. In EMPA-REG OUTCOME, inclusion criteria dictated that all patients should have overt CVD, either as a diagnosis of CAD or a history of MI or stroke [7]. The CANVAS Program and DECLARE-TIMI 58 also included such patients, but additionally enrolled patients with multiple CV risk factors, such as dyslipidaemia, smoking or hypertension [8, 9].

Subgroup analyses have shown that no significant reduction in CV death was seen in the CANVAS Program and DECLARE-TIMI even when considering only patients with baseline CVD [8, 9, 29]. Similarly, no significant reduction in CV death was seen in patients with prior MI in DECLARE-TIMI 58 [30] or patients with cerebrovascular disease in the CANVAS Program [31].

As a renal study rather than a CVOT, CREDENCE did not have any requirements for CV risk in patient enrolment, but nevertheless reported a 50% prevalence of CVD at baseline, and so included a sizeable cohort of at-risk patients [23]. An analysis of CV death in subgroups with and without baseline CVD has not been reported for this study; however, the primary outcome, which was a composite of CV death and several nephropathy outcomes, had a near identical response to treatment in both subgroups [23].

Therefore, current evidence suggests that differences in CVD prevalence between study cohorts cannot readily account for the reduction of CV death in EMPA-REG OUTCOME but not other SGLT2 inhibitor CVOTs. By including a broader CV risk population than EMPA-REG OUTCOME, future results from EMPRISE will provide important context to our understanding of CV death reductions with empagliflozin.

Similarly to CVD, renal disease is prevalent as a major mortality risk in patients with T2D [32, 33]. However, differences in renal populations also cannot convincingly explain the inconsistent CV death results. A renal subgroup analysis of EMPA-REG OUTCOME showed that the effect of empagliflozin on CV death did not increase with declining baseline renal function, suggesting that reduced renal function was not a key driver of treatment benefit [34]. A similar subgroup analysis of DECLARE-TIMI 58 also showed a lack of interaction between renal risk and CV death, with dapagliflozin producing a seemingly neutral effect in all renal risk groups [9], while the baseline renal risk profile of the CANVAS Program cohort was similar to EMPA-REG OUTCOME, and not linked to CV outcomes [34, 35]. More recently, the CREDENCE study, in which the majority of patients had reduced renal function, showed that canagliflozin narrowly missed statistical significance for reducing CV death even in this at-risk group of patients [23].

Therefore, CV death outcomes cannot easily be ascribed to differences in either CVD or renal profiles at baseline. As such, the class effect question remains a pertinent one, and any further light that future EMPRISE results can shed on CV death outcomes with empagliflozin will be of great interest.

### Guidelines

The first EMPRISE results [16] are consistent with recent guideline updates (Fig. 1a) [1, 3] in showing that empagliflozin may be preferred to DPP-4 inhibitors where the goal is to reduce HHF events. EMPRISE also supports the recent AHA and ACC recommendation [5] favouring SGLT2 inhibitors for primary prevention of HF in patients with T2D (Fig. 1c), although evidence from ongoing dedicated HF RCTs [28] may be required before other guidelines and reimbursement decisions will follow suit. Future insights from EMPRISE will provide welcome guidance on the implications of empagliflozin vs DPP-4 inhibitor use in patients for whom guidelines do not express a preference, including real-world effectiveness patterns and savings in healthcare resource utilisation and cost.

## Future data releases—what to look for in safety and health economic outcomes

### Safety

While the safety profile of empagliflozin in clinical studies has been generally favourable [7, 36], rare events may only be detectable in a larger cohort, such as that provided by EMPRISE. This will add to reassurances on safety with SGLT2 inhibitors in routine clinical practice provided by previous RWE studies, such as the CVD-REAL programme, which compared SGLT2 inhibitors as a class with all other glucose-lowering drugs [24–26]. For example, CVD-REAL has shown that SGLT2 inhibitors do not increase the risk of MI and stroke outcomes, consistent with CVOT findings [37]. Accumulating evidence for rare events of DKA with SGLT2 inhibitors [23, 29] has led to a warning on product labels advising alertness for this potentially dangerous event, and its atypical presentation in patients with only moderately increased blood glucose [38]. By study completion, EMPRISE should provide evidence from a cohort of 200,000 patients [16] to improve our understanding of this rare event and which patients are most at risk. Furthermore, confirming the safety profile of empagliflozin vs DPP-4 inhibitors in the diverse patients seen in clinical practice will reassure clinicians who are seeking a suitable alternative to DPP-4 inhibitors in a CVD setting.

### Healthcare resource utilisation

For many of us, the barrier posed by reimbursement requirements is a major driver of treatment choice. We expect that reducing HHF with SGLT2 inhibitors has the potential to generate resource and cost savings to health systems, as hospitalisation is responsible for a substantial proportion of the lifetime costs of HF management [39]. We look forward to EMPRISE results on health economic outcomes that will quantify these savings for various aspects of resource utilisation.

## Conclusions

We are commonly faced with a treatment choice between SGLT2 inhibitors and DPP-4 inhibitors in the management of T2D, in patients with a spectrum of CV risk. Early results from EMPRISE can already begin to inform such treatment choices, confirming that the HHF benefit with empagliflozin in EMPA-REG OUTCOME translates to a comparison with DPP-4 inhibitors in a real-world population. Future data releases will provide additional insights on other effectiveness outcomes, as well as safety and health economic outcomes.

While RWE is inevitably limited by the possibility of residual confounding [16], we welcome the careful attention paid by the authors of EMPRISE to minimise possible sources of bias [16], such as the steps taken to address concerns relating to immortal time bias that have been raised with previous RWE studies [19–21], providing more confidence in its results.

The role of RWE in complementing CVOTs is increasingly being recognised, including by regulatory bodies such as the US FDA, NICE in the UK, and the German IQWiG [10]. Despite the unavoidable limitations associated with RWE studies, the advantages of a cohort of 200,000 patients cannot be denied, and such a large-scale study would not be feasible under RCT conditions. It is hoped that this large cohort will provide additional context to CVOT-generated insights into CV protection with empagliflozin.

## Data Availability

Not applicable.

## Abbreviations

ACC: American College of Cardiology
ADA: American Diabetes Association
AHA: American Heart Association
CAD: coronary artery disease
CKD: chronic kidney disease
CV: cardiovascular
CVD: CV disease
CVOT: CV outcomes trial
DKA: diabetic ketoacidosis
DPP-4: dipeptidyl peptidase-4
EASD: European Association for the Study of Diabetes
FDA: Food and Drug Administration
GLP-1 RA: glucagon-like peptide-1 receptor agonist
HF: heart failure
IQWiG: Institut für Qualität und Wirtschaftlichkeit im Gesundheitswesen
MI: myocardial infarction
NICE: National Institute for Health and Care Excellence
PAD: peripheral artery disease
PS: propensity score
RCT: randomised controlled trial
RWE: real-world evidence
SGLT2: sodium–glucose transporter 2
T2D: type 2 diabetes
